# Effects of rehabilitation training on an elderly population with mild to moderate hearing loss: study protocol for a randomised clinical trial

**DOI:** 10.12688/f1000research.23332.3

**Published:** 2020-09-15

**Authors:** Parisa Rasouli Fard, Farnoush Jarollahi, Seyyed Jalal Sameni, Mohammad Kamali

**Affiliations:** 1 Department of Audiology, School of Rehabilitation Sciences, Iran University of Medical Sciences, Tehran, 15459-13487, Iran

**Keywords:** Age related hearing loss, Presbycusis, Temporal Fine Structure, Rehabilitation Training

## Abstract

**Background:** Age-related hearing loss (presbycusis) is a form of hearing loss in over 60-years-olds and has a negative impact on quality of life. Presbycusis is multifactorial and is predominately characterised with a loss of speech perception in noise. In the cochlea, auditory filters decompose broadband sound into a series of narrowband output signals, which contains two kinds of temporal information: slow changes in overall amplitude envelope (ENV) and faster variations in temporal fine structure (TFS). TFS is important for recognition of target speech in noise. The main aim of the study is to evaluate the effect of TFS rehabilitation training in participants over the age of 60 years with mild to moderate hearing loss.

**Methods:** A randomised clinical trial  conducted on 30 participants with mild (loss of 20-39dB HL) to moderate (40-69dB HL) hearing loss, aged between 60 and 75 years old. Participants with conductive hearing loss, abnormal middle ear pathology and central nervous system disease were excluded. Participants were selected randomly  to an intervention and control group with a 1:1 ratio. Rehabilitation for the intervention Group are 30-minute sessions three times a week for a total five weeks of vowel consonant vowel words that are used to eliminate ENV and keep only TFS. Word in noise test, binaural TFS test, and Speech, Spatial and Qualities of Hearing Scale scores are performed at the beginning and end of study to evaluate the effect of rehabilitation training.

**Conclusion: ** Life expectancy in the elderly has improved, leading to an increased prevalence of age-related diseases including presbycusis. A literature review highlighted that TFS damage is permanent; however, in this study we will attempt to prove that TFS training may lead to speech in noise perception improvement.

**Trial registration: **Registry of Clinical Trials, IRCT2019625044006N1 (7 ^th^ August 2019).

## Introduction

Presbycusis (age-related hearing loss) is one of the most common disorders worldwide ^1, 2^. The cause of presbycusis is multifactorial, including pathophysiological degeneration, extrinsic and intrinsic damage, genetic predisposition and comorbidities (conditions like diabetes, hypertension and stroke) ^3– 5^.

In cochlea high frequency sounds evoke greatest vibration of the basilar membrane at the base while lower frequency sounds evoke greatest vibration at the apex ^6– 9^. Sounds are decomposed to narrow band signals temporal envelope (ENV) and rapid oscillations temporal fine structure (TFS) ^9– 12^. The ENV frequency range is between 2–50 Hz. One of the most important tasks of ENV is to identify speech in quiet environments ^11, 12^. TFS frequency range is between 0.6–10 kHz ^13^, and TFS cues are important in perception of pitch, tone separation ^14^, and identify target speech in interfering sounds ^15^. Presbycusis is associated with loss of speech perception in noisy environments ^16^ and deterioration of the processing of TFS information ^14, 15, 17^.

Previous studies indicate that sensorineural hearing loss is associated with a reduction in speech recognition and is dependent on deterioration of TFS ^18^, showing the importance of TFS for listening with background sounds ^8^. Studies by Hopkins *et al.* suggest that TFS is important to recognise the temporal dips in fluctuating background noise ^19, 20^. In an elderly population with high frequency hearing loss, even when absolute thresholds are within the normal range, the TFS can be damaged ^21^. It is speculated that TFS information is useful for separation of the target speech in background speech ^22^.

### Objectives

The main aim of the study is to evaluate the impact of special rehabilitation training based on TFS on improvement of speech in noise perception in an elderly population with mild to moderate hearing loss.

## Protocol

This is version 3 of the protocol. There is no plan for further trial modifications.

### Study overview

We conduct a randomised clinical trial of rehabilitation training on speech in noise perception performance on an elderly population with mild to moderate hearing loss at the Audiology Clinic of School of Rehabilitation Sciences, Iran University of Medical Sciences (Tehran, Iran). It is hypothesized that the inability to use TFS speech cues is the main cause of speech perception problem in noise in elderly individuals, and it is possible by designing appropriate rehabilitation exercises to reduce the difficulty of speech perception in noise.

The Medical Ethics Committee at the Iran University of Medical Sciences approved the registered study protocol (IR.IUMS>REC.1398.003). The study was registered on the Iranian Registry of Clinical Trials (registration number, IRCT2019625044006N1), a Primary Registry in the World Health Organization Clinical Trials Registry Network.

The protocol does not involve complications for participants in the study. All participants were informed both verbally and in writing about the study procedure. Written consent to participants were obtained from the participants before the study start (see *Extended data:* S1).

### Terminology used in this study

*Mild to moderate hearing loss:* auditory thresholds ≤25dB within the frequency<2000 Hz and 25–70 dB HL with frequency 2000–8000 Hz.

*TFS-LF test:* software designed by Hopkins and Moore in 2010. The test is originally based on measuring the interaural phase differences ^15^.

*Interaural phase difference (IPL):* lowest difference in the phase of the wave in each ear and dependent frequency sound waves and difference in time between ears ^15^.

*Signal to noise ratio (SNR):* ratio of the power of a signal (meaningful information) to the power of background noise (unwanted signal), expressed in decibels (dB). Larger numbers for signal characteristics mean better and more useful than unwanted noise information ^23^. In this study the signal-to-noise ratio levels were 0, 4, 8, 12, 16, 20, and 24 dB.

*Speech in noise score:* measured by Persian version of WIN test (PARWIN list), which is expressed as a percentage by performing a single syllable word test. The PARWIN test is a version of the Richard H. Wilson WIN test, in which the background noise in this test is baffled noise ^24^. PARWIN test is used to estimate SNR (50%) using Spearman Karber equation.

*Speech, Spatial and Qualities of Hearing Scale (SSQ) questionnaire:* used in previous studies in elderly individuals with communication disorders caused by hearing loss. From the original version of the SSQ questionnaire, its validity and reliability native version were confirmed (validity, 96% reliability) and included 47 items in three subgroups of speech perception, spatial hearing, and auditory quality. Based on the results of the questionnaire, the mean score of each item and item of each index will be measured for the research participants.

*Rehabilitation training:* auditory rehabilitation was based on TFS. The intervention group will be asked to identify vowel consonant vowel words (VCVs) that have only TFS preserved and their envelope discarded. It is based on that VCVs that processing and converting to TFS speech. In this process the ENV of VCVs are eliminated and only TFS will be kept.

### Participants

Participants were recruited from elderly people, aged between 60 and 75 years old, referred to the audiology clinics of Iran University of Medical Sciences and were informed by phone about the study. They were selected based on previous clinical examination, including otoscopy, tympanometry and pure tone audiometry test (PTA) to identify type and level of a hearing loss. In a preliminary interview, speech perception difficulty was evaluated with a question if they had difficulty in understanding speech in noise. Those who respond yes were entered into the study. Later to define difficulty in understanding speech in noise we adapted questionnaire which was used by Tokgoz-Yilmaz *et al*. ^25^.

We performed Mini Mental State Examination (MMSE) questionnaire in order to rule out prominent cognition difficulty in participants.

Participants were informed that can withdraw from the study at any time. Privacy concerning information and results of participants are respected.

The schematic diagram of study procedures is shown below *Extended data:* Figure S1.

***Inclusion and exclusion criteria***. *Inclusion criteria:* individuals with mild to moderate hearing loss and aged between 60–75 years, having diploma or higher degree; right-handedness (assessed using Edinburgh handedness inventory); speaking native language and being monolingual; complaint about speech in noise perception difficulties and normal condition of middle ear function.

*Exclusion criteria:* those who do not meet the inclusion criteria, unwillingness for participation in each step of study, conductive hearing loss and abnormal middle ear, central nervous system disease, head trauma, history of seizure attack and epilepsy, and use of psychiatric and nervous system drugs. Individuals with obvious cognitive problems, as diagnosed by Mini Mental State Examination (MMSE), were also excluded.

***Sample size***. The following formula is used to determine the number of samples in each group with the concern that the two groups are independent and dependent variables in this study are quantitative.

$$\text{n}=\frac{{\left(Z_{1-\frac{\text{α}}{2}}+Z_{1-\text{β}}\right)}^{2}({\sigma_{1}}^{2}+{\sigma_{2}}^{2})}{{\left({\text{μ}}_{1}-{\text{μ}}_{2}\right)}^{2}}$$

α1: standard deviation of the studied variable in the first group (case, exposed, or intervened)

α2: standard deviation of the studied variable in the second group (control, unexposed, or compared)

μ1: mean of the studied variable in the first group

μ2: mean of the studied variable in the second group

α=0.05

β=80%

Z= 1.96

Based on previous studies, a power of 85% and level of significance of 95% was determined for this study. We obtained a sample size of 15 individuals for each group (total = 30), which takes into consideration a 20% drop out.

### Study design

The study will not involve complications for participants, but if there is extreme difficulty with cooperation for participants the test will be discontinued. All participants were informed both orally and in writing about the study process. Written consent to participant were obtained before the study start. There is no criteria for intervention modification in this study protocol. To improve adherence to intervention protocols, every training session the examiner provide feedback to all participants and inform them about the training progress. The rehabilitation sessions and duration are flexible for participant.

We randomly assigned participants in 1:1 ratio, intervention and control group. The intervention group undergo the rehabilitation training program.

The two groups were matched for age and gender. Those in the control group do not receive any rehabilitation programs during the study. The randomization was applied by random number table (those assigned an odd number, control group; those assigned an even number, intervention group).

***Study procedures***. Pre-rehabilitation, the SNR (50%) of all participants was measured using the word in noise (WIN) test. In addition, a binaural TFS test and the SSQ questionnaire score of all participants are evaluated (see section *Outcomes* below).

***Rehabilitation training***. Participants identify the set of 16 consonants using one-interval forced-choice procedure and feedback with correct answer. On each test the participants select one of the stimuli from the set of 16 syllables. The participants are informed while the stimulus is presented that they should identify its middle consonant. Following each stimulus presentation, a 4 × 4 visual display of the response alternatives appear on a computer monitor and the participant select the response by using the computer mouse.

Each participant select a box, if they click the box correctly, the box turn green and if they choose the wrong answer, the box turn red. The participant is given visual feedback by showing the correct VCV with a yellow box. No time limit is be imposed on the participant's responses. Each experimental run consists of 64 trials derived from a different random-order presentation of the 64 syllables in the stimulus set. Each run is last 16 to 30 min depending on the participant's response time. The total duration of rehabilitation sessions are e five weeks. Experiments are controlled by a desktop PC.

Only the intervention group undergo the rehabilitation and control group is not informed about details of the intervention study procedure.

### Speech stimulus process

The TFS speech consists of single syllable recorded in / a / C / a / with various 16 consonant format which included Aja, Aka, Ara,…. and it is pronounced by a native-speaking man. The analogue signals are converted to digital a 16-bit at 44.1 KHz sampling frequency. The stimulus synthesis process is performed in MATLAB software and the software is provided in C programming language.

The original bandpass is filtered into 16 bands of equal bandwidth on a log frequency scale spanning 80 to 8020 Hz. Each Bandpass signal is decomposed to ENV and TFS by Hilbert transform. The ENV component is discarded and TFS component is normalized and TFS component in each band summed lastly creating TFS speech.

After rehabilitation, the SNR (50%) using the WIN test, and a binaural TFS test and SSQ questionnaire will be evaluated again in intervention group. Results will be compared in intervention and control groups before and after the rehabilitation program.

***Outcomes***. *SNR (50%):* single syllable words in the presence of noise at different signal-to-noise ratios (0, + 4, + 8, + 12, + 16, + 20, + 24) as binaural in two study groups and compare the SNR (50%). Differences in scores before and after rehabilitation training between the two groups will be compared.

The Words-in-Noise (WIN) materials were developed to evaluate the ability of listeners to understand words in multitalker babble. The WIN involves in which the level of the noise is fixed and five words are presented at seven signal-to-noise ratios from 24 to 0 dB in 4 dB decrements. The 35 words are spoken by a native male speaker. The metric of interest is the signal-to-noise ratio (S/N) at which recognition performance is 50%, which is a value determined with the Spearman Karber equation see ( *Extended data:* S2).

Binaural TFS test: determines the binaural change of phase difference at different frequencies in intervention and control groups before and after rehabilitation training.

The correlation between the results of speech perception test scores in the presence of noise with the results of binaural TFS test in the two groups after rehabilitation program will be assessed.

*SSQ questionnaire score:* provided to each group before and after rehabilitation training. Scores between intervention and control groups will be compared ( *Extended data:* S3).

### Statistical analysis

In descriptive analysis of data, central tendency and dispersion indices (mean, median and standard deviation) will be used. Kolmogorov–Smirnov test will be used to test whether two random samples are drawn from the same normal distribution. Otherwise its nonparametric equivalent will be used. Depending on the circumstances, paired t-test and analysis of covariance will be used to compare pre- and post-rehabilitation program. Other analytical tests will be used as required during the data processing phase. SPSS software (V20.0, IBM Corporation, New York, USA) will be used for statistical data analysis and the significance level for all tests will be 0.05.

### Dissemination

The results of our research will be disseminated through presentations at regional and national audiology conferences. The study outcomes will be published through peer-reviewed journals. There is no limit in the publication of the trial results.

### Monitoring

Eight independent audiology experts who are the academic members of rehabilitation schools in Shahid Beheshti University of Medical Sciences (SBMU) and Iran University of Medical Sciences (IUMS) will monitor patient safety and treatment efficacy. They approved the relevance, clarity and simplicity material of the study.

## Study status

The enrolment of the patients has been performed and the allocation will be performed in the near future. The study started in November 2019 and will continue until December 2020.

## Discussion

Elderly populations are growing rapidly worldwide, and this higher number of older individuals is associated with an increase in prevalence and incidence of age-related disorders. Age-related hearing loss (presbycusis) is one the most common disorders with an increase in age. Hearing loss has significant negative impact in quality of life in elderly population. It causes limitation of communication and social activity ^1^.

Speech perception in noisy environments is very serious difficulty with presbycusis, The presbycusis is known as damage of hair cells in cochlea ^5^. The hair cells damage with presbycusis mostly associated with deteriorate of temporal fine structure (TFS) information ^7^.

TFS is important when background sounds are present ^9^. Loss of speech perception in noisy environments with presbycusis is mostly caused by damage of processing of TFS information. Our study is based on improvment of TFS deficit in cochlea.

The rehabilitation training is based on identify vowel consonant vowel words (VCV). 

The test is performed in several sessions. The total time of test is three times every week for total fives week for all subjects but the time in every session is different between participants.

To evaluate the efficiency of rehabilitation training we used temporal fine structure sensitivity test (TFS test), Speech in noise test (SNR 50%) and Speech, Spatial and Qualities of Hearing Scale (SSQ) 

They are performed in both intervention and control group but at the end of study we measure only the tests in intervention population.

Initially TFS1 was developed by Moore and Sek (2009) in order to discriminate a harmonic complex tone from the same tones with all components shifted upwards by the same amount in hertz leading to an inharmonic tone. The TFS-LF was described by Moore and Hopkins (2010) to measuring thresholds for detecting changes in interaural phase difference ( IPD). Although the TFS-LF is used generally but it has a limitation. The frequency of the tones is fixed, for example at 250, 500 or 750 Hz and some old listener have difficulty to perform the test with chosen frequency. This limitation led to develop TFS-AF test. In TFS-AF test IPD is fixed for example is 180 degree with frequency adaptively varied to determine a threshold. In this study, we used TFS-LF test in all participants because TFS-LF test provides a good measure of sensitivity to binaural TFS. The aim of this study was to improve speech in noise perception with fast transition consonant vowel words (VCV) and identify interaural phase difference (IPD) which TFS-LF is more appropriate. TFS-LF were performed in three frequencies, 250, 500 and 750 Hz ^15^. 

Signal-To-Noise Ratio (SNR) 50% is used routinely by audiology clinics. Age related hearing loss have decline speech discrimination ability in similar SNR compare to normal hearing ability and require higher SNR ^23^.

Several test and questionnaire are performed to assess the ability to hear and process sounds and speech from different locations which called spatial hearing speech. In this study we use The Speech, Spatial and Qualities of Hearing Scale (SSQ).

The validity of TFS-LF, SNR 50% and SSQ questionnaire to recognise hearing loss have been confirmed previously ^15, 22, 23^.

We designed stimulus software which divided sound signal to envelope (ENV) and TFS. In this program ENV is removed and only TFS is preserved.

In conclusion in this study, we will attempt to prove by special rehabilitation training based on TFS damage age-related hearing loss can be improved.

## Data availability

### Underlying data

No data is associated with this article.

### Extended data

Open Science Framework: Effect of rehabilitation training on an elderly population with mild to moderate hearing loss: study protocol for a randomised clinical trial, https://doi.org/10.17605/OSF.IO/VU9CH ^26^.

This project contains the following extended data:

- S1: Questionnaire.
- S2: Words-in-Noise (WIN) test to measure single syllable words (SNR) 50%
- S3: Speech, Spatial and Qualities of Hearing Scale (SSQ) questionnaire
- Figure S1: Schematic diagram of study procedures and timeline ****

Open Science Framework: Effect of rehabilitation training on an elderly population with mild to moderate hearing loss: study protocol for a randomised clinical trial, https://doi.org/10.17605/OSF.IO/A4KGM, registered on 1 ^st^ June 2020 ^26^.

This project contains the following extended data:

- *Informed consent: Form has been uploaded to OSF https://osf.io/a4kgm*

### Reporting guidelines

Open Science Framework: SPIRIT checklist for ‘Effect of rehabilitation training on an elderly population with mild to moderate hearing loss: study protocol for a randomised clinical trial’, https://doi.org/10.17605/OSF.IO/VU9CH ^26^.

Data are available under the terms of the Creative Commons Zero "No rights reserved" data waiver (CC0 1.0 Public domain dedication).
