# Supplementary material for: Effects of rehabilitation training on an elderly population with mild to moderate hearing loss: study protocol for a randomised clinical trial
Source: F1000Res. 2020 Sep 15;9:582. Originally published 2020 Jun 9. [Version 3] doi: 10.12688/f1000research.23332.3 (PMC7416574; doi:10.12688/f1000research.23332.3)
Supplement: Peer review history

# Peer review: Reviewer response for version 3

**DOI:** 10.5256/f1000research.29137.r71385

**Published:** 2020-09-25

**Authors:** Ali Danesh

**Affiliations:** 1 Department of Communication Sciences and Disorders, Florida Atlantic University, Boca Raton, FL, USA

1. In Abstract and other sections please change intervention to interventional, Group to group, end to "the end".
2. In terminology, please always use dB HL instead of dB! Also, in "Terminology used in this study" include the definition of TFS. TFS definition must also be included in the introduction.
3. Change " Participants can withdraw from the study at any time. Privacy concerning information and results of participants are respected." To Participants were instructed that they can withdraw from the study at any time. Privacy concerning information and results of participants are respected."
4. A paragraph cannot be only one sentence such as" They are performed in both intervention and control group but at the end of study we measure only the tests in intervention population." Please combine with the previous portions.
5. Change the discussion to the following format:

Elderly populations are growing rapidly worldwide, and this higher number of older individuals is associated with an increase in prevalence and incidence of age-related disorders. Age-related hearing loss (presbycusis) is one the most common disorders with an advancing age. Hearing loss has significant negative impact on quality of life in the elderly population and it may result in isolation and memory loss. It causes limitation of communication and social activity 1 . Speech perception in noisy environments is a very serious difficulty with presbycusis.  Presbycusis is known as a damage of hair cells in the cochlea 5. Hair cells damage with presbycusis mostly is associated with deterioration of temporal fine structure (TFS) information 7 . TFS is important when background sounds are present 9 . Loss of speech perception in noisy environments with presbycusis is mostly caused by damage to the processing of TFS information. Our study is based on the restoration of TFS deficit in cochlea. The proposed rehabilitation training is based on identifying the vowel consonant vowel words (VCV). It is known that hearing loss is associated with difficulties in recognising VCV words. Improvement of speech perception in noise is related to better recognition of VCV in hearing impaired people. The study procedural protocol was performed in several sessions and the total of number of the sessions was three times every week for a total of five weeks for all subjects; however, the time in every session was different between participants depending on their needs.

 In order to evaluate the efficiency of rehabilitation training we used temporal fine structure sensitivity test (TFS test), Speech in Noise Test (SNR of 50%) and Speech, Spatial and Qualities of Hearing Scale (SSQ) test.  They were performed in both interventional and control group but at the end of study we measured only the tests in interventional group. The TFS test was introduced by Moore and Sek (2009) to discriminate harmonic complex tone from inharmonic tone. In this study we used TFS-LF test but later due to difficulty of TFSLF in some listeners TFS-LF was modified to TFS-AF test 15 . Signal-to-Noise Ratio (SNR) 50% is used routinely by audiology clinics. Age related hearing loss showed decline speech discrimination ability in similar SNR compared to normal hearing ability and requires higher SNR 23 . Several tests and questionnaires were performed to assess the ability to hear and process sounds and speech from different locations which is called spatial hearing speech. In this study we used the Speech, Spatial and Qualities of Hearing Scale (SSQ). The validity of TFS-LF, SNR 50% and SSQ questionnaire to recognise hearing loss have been confirmed previously 15, 22, 23 . We designed stimulus software which divided sound signal to envelope (ENV) and TFS. In this program ENV is removed and only TFS is preserved. In conclusion in this study, we attempted to prove that by special rehabilitation training based on TFS damage age-related hearing loss can be re-established.

 I think you need to expand the discussion but I vote to approve your manuscript as submitted, after the above corrections have been made.

Is the study design appropriate for the research question?

Yes

Is the rationale for, and objectives of, the study clearly described?

Yes

Are sufficient details of the methods provided to allow replication by others?

Partly

Are the datasets clearly presented in a useable and accessible format?

Partly

Reviewer Expertise:

NA

I confirm that I have read this submission and believe that I have an appropriate level of expertise to confirm that it is of an acceptable scientific standard.

# Peer review: Reviewer response for version 3

**DOI:** 10.5256/f1000research.29137.r71384

**Published:** 2020-09-21

**Authors:** Mojtaba Tavakoli

**Affiliations:** 1 Department of Audiology, School of Rehabilitation, Ahvaz Jundishapur University of Medical Sciences, Ahvaz, Iran

No further comment.

Is the study design appropriate for the research question?

Yes

Is the rationale for, and objectives of, the study clearly described?

Yes

Are sufficient details of the methods provided to allow replication by others?

Yes

Are the datasets clearly presented in a useable and accessible format?

Yes

Reviewer Expertise:

Hearing aid

I confirm that I have read this submission and believe that I have an appropriate level of expertise to confirm that it is of an acceptable scientific standard.

# Peer review: Reviewer response for version 2

**DOI:** 10.5256/f1000research.28485.r69198

**Published:** 2020-08-17

**Authors:** Suna Tokgoz‐Yilmaz

**Affiliations:** 1 Diagnosis‐Rehabilitation Center of Hearing, Balance and Speech‐Language Disorders, Ankara University School of Medicine, Ankara, Turkey

Excellent study, but regarding a topic you mentioned in the research

 "The TFS test was present by Moore and Sek 2009 to discriminate harmonic complex tone from inharmonic tone. In this study we used TFS-LF test but later due to difficulty of TFS-LF in some listeners TFS-LF was modified to TFS-AF test ^15^."

- "due to difficulty of TFS-LF in some listeners".....what difficulties and will the TFS-AF test be applied to all participants?
- If both TFS-AF and TFS-LF tests will be applied, what will be the number of participants?
- How will this be decided?

wish you good work.

Is the study design appropriate for the research question?

Yes

Is the rationale for, and objectives of, the study clearly described?

Yes

Are sufficient details of the methods provided to allow replication by others?

Partly

Are the datasets clearly presented in a useable and accessible format?

Partly

Reviewer Expertise:

Audiology

I confirm that I have read this submission and believe that I have an appropriate level of expertise to confirm that it is of an acceptable scientific standard, however I have significant reservations, as outlined above.

# Response

**Published:** 2020-08-18

**Authors:** Parisa Rasouli Fard

**Affiliations:** Department of Audiology, School of Rehabilitation Sciences, Iran University of Medical Sciences. Tehran, Iran, Iran

Dear Dr. Tokgöz Yılmaz,

 Thank you very much for important comment.

*Initially TFS1 was developed by Moore and Sek (2009) in order to discriminate a harmonic complex tone  from the same tones with all components  shifted upwards by the same amount in hertz leading to an inharmonic tone (1) .*

*The TFS-LF was described by Moore and Hopkins (2010) to measuring thresholds for detecting  changes in interaural phase difference ( IPD) (2) .  Although the TFS-LF is used generally but it has a limitation. The frequency of the tones is fixed, for example at 250, 500 or 750 Hz and some old listener have difficulty to perform the test with chosen frequency.*

*This limitation led to develop TFS-AF test. In TFS-AF test IPD is fixed for example is 180 degree with frequency adaptively varied to determine a threshold. (3).*

 In this study, we used TFS-LF test in all participants because TFS-LF test provides a good measure of sensitivity to binaural TFS. The aim of this study was to improve speech in noise perception with fast transition consonant vowel words (VCV) and identify interaural phase difference (IPD) which TFS-LF is more appropriate. TFS-LF were performed in three frequencies, 250, 500 and 750 Hz.

 Four participants (three interventional and one ion control group) needed more time to perform TFS-LF test.

 Best wishes

 1.                        Moore BC, Vickers DA, Mehta A. The effects of age on temporal fine structure sensitivity in monaural and binaural conditions. Int J Audiol. 2012;51(10):715-21.

 2.                        Moore BC, Sek A. Development of a fast method for determining sensitivity to temporal fine structure. Int J Audiol. 2009;48(4):161-71.

 3.                        Fullgrabe C, Harland AJ, Sek AP, Moore BCJ. Development of a method for determining binaural sensitivity to temporal fine structure. Int J Audiol. 2017;56(12):926-35.

# Peer review: Reviewer response for version 2

**DOI:** 10.5256/f1000research.28485.r69197

**Published:** 2020-08-13

**Authors:** Mojtaba Tavakoli

**Affiliations:** 1 Department of Audiology, School of Rehabilitation, Ahvaz Jundishapur University of Medical Sciences, Ahvaz, Iran

All the comments have been done .

Is the study design appropriate for the research question?

Yes

Is the rationale for, and objectives of, the study clearly described?

Yes

Are sufficient details of the methods provided to allow replication by others?

Yes

Are the datasets clearly presented in a useable and accessible format?

Yes

Reviewer Expertise:

Hearing aid

I confirm that I have read this submission and believe that I have an appropriate level of expertise to confirm that it is of an acceptable scientific standard.

# Peer review: Reviewer response for version 1

**DOI:** 10.5256/f1000research.25755.r65520

**Published:** 2020-08-07

**Authors:** Ali Danesh

**Affiliations:** 1 Department of Communication Sciences and Disorders, Florida Atlantic University, Boca Raton, FL, USA

Please note that each comment is prefixed by AAD and a number. You can see the comments at the end of this report. The report is also attached as a file here.

 Thank you.

**Effect**[AAD1] **of rehabilitation training on an elderly population with mild to moderate hearing loss: study protocol for a randomised clinical trial**

  [version 1; peer review: 1 approved]

 Parisa Rasouli Fard[AAD2] 

  https://orcid.org/0000-0003-4985-435X

 , Farnoush Jarollahi, Seyyed Jalal Sameni, Mohammad Kamali

 Author details

Abstract

**Background:** Age-related hearing loss (presbycusis) is a form of hearing loss in over 60-year-olds and has a negative impact on quality of life. The pathophysiology of presbycusis is multifactorial and is predominately characterised with a loss of speech perception in noise[AAD3] . In the cochlea, auditory filters decompose broadband sound into a series of narrowband output signals, which contains two kinds of temporal information: slow changes in overall amplitude envelope (ENV) and faster variations in temporal fine structure (TFS). TFS is important for recognition of target speech in noise. The main aim of the study is to evaluate the effect of TFS rehabilitation training in participants over the age of 60 years with mild to moderate hearing loss.

**Methods:** A randomised clinical trial will [AAD4] be conducted on 30 participants with mild (loss of 20-39dB[AAD5] ) to moderate (40-69dB[AAD6] ) hearing loss, aged between 60 and 75 years old. Participants with conductive hearing loss, abnormal middle ear pathology and central nerve[AAD7]  system disease will[AAD8]  be excluded. Participants will [AAD9] be randomly selected to an intervention and control group with a 1:1 ratio. Rehabilitation for the intervention group will be 30-minute sessions three times a week for a total five weeks of vowel consonant vowel words that are used to eliminate ENV and keep only TFS. Word in noise test, binaural TFS test, and Speech, Spatial and Qualities of Hearing Scale scores will be performed at the beginning and end of study to evaluate the effect of rehabilitation training.

**Conclusion:**[AAD10]   Life expectancy in the elderly has improved, leading to an increased prevalence of age-related diseases including presbycusis. A literature review highlighted that TFS damage is permanent; however, in this study we will attempt to prove [AAD11] that TFS training may lead to speech in noise perception restored.[AAD12]

**Trial registration: **Registry of Clinical Trials, IRCT2019625044006N1 (7 ^th^ August 2019).

Keywords

Age related hearing, Presbycusis, Temporal Fine Structure, Rehabilitation Training[AAD13]

Corresponding author: Farnoush Jarollahi

Competing interests: No competing interests were disclosed.

Grant information: This study was part of a Ph.D. Dissertation approved by Iran University of Medical Sciences (IUMS), Tehran, Iran and is financially supported by IUMS (Contract No: 98-1-6-14345).

*The funders *[AAD14] *had no role in study design, data collection and analysis, decision to publish, or preparation of the manuscript.*

Copyright:  © 2020 Rasouli Fard P *et al*. This is an open access article distributed under the terms of the Creative Commons Attribution License, which permits unrestricted use, distribution, and reproduction in any medium, provided the original work is properly cited.

How to cite: Rasouli Fard P, Jarollahi F, Sameni SJ and Kamali M. Effect of rehabilitation training on an elderly population with mild to moderate hearing loss: study protocol for a randomised clinical trial [version 1; peer review: 1 approved]. *F1000Research* 2020, 9:582 ( https://doi.org/10.12688/f1000research.23332.1)First published: 09 Jun 2020, 9:582 ( https://doi.org/10.12688/f1000research.23332.1)Latest published: 09 Jun 2020, 9:582 ( https://doi.org/10.12688/f1000research.23332.1)

Introduction

Presbycusis (age-related hearing loss) is one the most common disorders worldwide 1, 2. The cause of presbycusis is multifactorial, including pathophysiological degeneration, extrinsic and intrinsic damage, genetic predisposition and comorbidities (conditions like diabetes, hypertension and stroke) ^3– 5^.

In cochlea high frequency sounds evoke greatest vibration of the basilar membrane at the base while lower frequency sounds evoke greatest vibration at the apex 6– 9. Sounds are decomposed to narrow band signals (envelope; ENV)[AAD15]  and rapid oscillations (temporal fine structure; TFS[AAD16] ) ^9– 12^. The ENV frequency range is between 2-50 Hz. One of the most important task o[AAD17] f ENV is to identify speech in quiet environments ^11, 12^. TFS frequency range is between 0.6-10 kHz ^13^, and TFS cues are important in perception of pitch, tone separation ^14^, and identify target speech in interfering sounds ^15^. Presbycusis is associated with loss of speech perception in noisy environments ^16^ and deterioration of the processing of TFS information ^14, 15, 17^.

Previous studies indicate that sensorineural hearing loss is associated with a reduction in speech recognition and is dependent on deterioration of TFS 18, showing the importance of TFS for listening with background sounds ^8^. Studies by Hopkins *et al.* suggest that TFS is important to recognise the temporal dips in fluctuating background noise ^19, 20^. In an elderly population with high frequency hearing loss, even when absolute thresholds are within the normal range, the TFS can be damaged ^21^. It is speculated that TFS information is useful for separation of the target speech in background speech ^22^.

Objectives

The main aim of the study is to evaluate the impact of special rehabilitation training based on TFS on improvement of speech in noise perception in an elderly population with mild to moderate hearing loss.

Protocol

This is version 1 of the protocol. There is no plan for further trial modifications.[AAD18]

Study overview

We will conduct a randomised clinical trial of rehabilitation training on speech in noise perception performance on an elderly population with mild to moderate hearing loss at the Audiology Clinic of School of Rehabilitation Sciences, Iran University of Medical Sciences (Tehran, Iran). It is assumed [AAD19] that the inability to use TFS speech cues is the main cause of speech perception problem in noise in elderly individuals, and it is possible by designing appropriate rehabilitation exercises to reduce the difficulty of speech perception in noise.

The Medical Ethics Committee at the Iran University of Medical Sciences approved the registered study protocol (IR.IUMS>REC.1398.003). The study was registered on the Iranian Registry of Clinical Trials (registration number, IRCT2019625044006N1), a Primary Registry in the World Health Organization Clinical Trials Registry Network.

The protocol does not involve complications for precipitants [AAD20] in the study. All participants will be informed both orally[AAD21]  and writing [AAD22] about the study process[AAD23] . Written consent to participant will be [AAD24] obtained from the participants before the study start (see *Extended data:* S1).

Terminology used in this study

*Mild to moderate hearing loss:* auditory thresholds ≤25dB [AAD25] within the frequency<2000 Hz and 25-70 dB with frequency 2000-8000 Hz.

*TFS-LF test:* software designed by Hopkins and Moore in 2010. The test is originally based on measuring the inter-aural phase differences 15.

*Inter aural *[AAD26] *phase difference (IPL):* lowest difference in the phase of the wave in each ear and dependent frequency sound waves and difference in time between ears 15.

*Signal to noise ratio (SNR):* ratio of the power of a signal (meaningful information) to the power of background noise (unwanted signal), expressed in decibels (dB). Larger numbers for signal characteristics mean better and more useful than unwanted noise information 23. In this study the signal-to-noise ratio levels were 0, 4, 8, 12, 16, 20, and 24 dB.

*Speech in noise score:* measured by PARWIN l[AAD27] ist, which is expressed as a percentage by performing a single syllable word test. The PARWIN test is a version of the Richard H. Wilson WIN test, in which the background noise in this test is baffled noise 24. PARWIN test is used to estimate SNR (50%) using Spearman Karber equation.

*Speech, Spatial and Qualities of Hearing Scale (SSQ) questionnaire:* used in previous studies in elderly individuals with communication disorders caused by hearing loss. From the original version of the SSQ questionnaire, its validity and reliability native version were confirmed (validity, 96% reliability) and included 47 items in three subgroups of speech perception, spatial hearing, and auditory quality. Based on the results of the questionnaire, the mean score of each item and item of each index will be measured for the research participants.

*Rehabilitation training:* auditory rehabilitation will be based [AAD28] on TFS. The intervention group will be asked to identify vowel consonant vowel words (VCVs) that have only TFS preserved and their envelope discarded. It is based on that VCVs that processing and converting to TFS speech. In this process the ENV of VCVs will be eliminated and only TFS will be kept.

Participants

Participants will be recruited [AAD29] from elderly people, aged between 60 and 75 years old, referred to the audiology clinics of Iran University of Medical Sciences and will be informed by phone about the study. They will be selected based on previous clinical examination, including otoscopy, tympanometry and pure tone audiometry test (PTA) to identify type and level of a hearing loss. In a preliminary interview, speech perception difficulty will be evaluated with a question if they had difficulty in understanding speech in noise. Those who respond yes will be entered into the study. We will perform Mini Mental State Examination (MMSE) questionnaire in order to rule out prominent cognition difficulty in participants.

Participants can[AAD30]  withdraw from the study at any time. Privacy concerning information and results of participants will be respected.[AAD31]

The schematic diagram of study procedures is shown below *Extended data:* Figure S1.

***Inclusion and exclusion criteria***. *Inclusion criteria:* individuals with mild to moderate hearing loss and aged between 60-75 years, having diploma or higher degree; right-handedness (assessed using Edinburgh handedness inventory); speaking native language and being monolingual; complaint about speech in noise perception difficulties and normal condition of middle ear function.

*Exclusion criteria:* those who do not meet the inclusion criteria, unwillingness for participation in each step of study, conductive hearing loss and abnormal middle ear, central nervous system disease, head trauma, history of seizure attack and epilepsy, and use of psychiatric and nervous system drugs. Individuals with obvious cognitive problems, as diagnosed by Mini Mental State Examination (MMSE), will also be excluded.

***Sample size***. The following formula is used to determine the number of samples in each group with the concern that the two groups are independent and dependent variables in this study are quantitative.

n=(Z1−α2+Z1−β)2(σ12+σ22)(μ1−μ2)2n=(Z1−α2+Z1−β)2(σ12+σ22)(μ1−μ2)2

α1: standard deviation of the studied variable in the first group (case, exposed, or intervened)

α2: standard deviation of the studied variable in the second group (control, unexposed, or compared)

μ1: mean of the studied variable in the first group

μ2: mean of the studied variable in the second group

α=0.05

β=80%

Z= 1.96

Based on previous studies, a power of 85% and level of significance of 95% was determined for this study. We obtained a sample size of 15 individuals for each group (total = 30), which takes into consideration a 20% drop out.

Study design

The study will not involve complications for participants, but if there is extreme difficulty with cooperation for participants the test will be discontinued. All participants will be informed both orally and in writing about the study process. Written consent to participant will be obtained before the study start. There is no criteria for intervention modification in this study protocol. To improve adherence to intervention protocols, every training session the examiner will provide feedback to all participants and will inform them about the training progress. The rehabilitation sessions and duration are flexible for participant.

We will randomly assign participants in 1:1 ratio, intervention and control group. The intervention group will undergo the rehabilitation training program.

The two groups will be matched for age and gender. Those in the control group will not receive any rehabilitation programs during the study. The randomization will be applied by random number table (those assigned an odd number, control group; those assigned an even number, intervention group).

***Study procedures***. Pre-rehabilitation, the SNR (50%) of all participants will be measured using the WIN[AAD32]  test. In addition, a binaural TFS test and the SSQ questionnaire score of all participants will be evaluated (see section *Outcomes* below).

***Rehabilitation training***. Participants will identify the set of 16 consonants using one-interval forced-choice procedure and feedback with correct answer. On each test the participants will select one of the stimuli from the set of 16 syllables. The participants will be informed while the stimulus is presented that they should identify its middle consonant. Following each stimulus presentation, a 4 × 4 visual display of the response alternatives will appear on a computer monitor and the participant will select the response by using the computer mouse.

The [AAD33] participant will select a box, if they click the box correctly, the box will turn green and if they chooses t[AAD34] he wrong answer, the box will turn red. The participant will be given visual feedback by showing the correct VCV with a yellow box. No time limit will be imposed on the participant's responses. Each experimental run consists of 64 trials derived from a different random-order presentation of the 64 syllables in the stimulus set. Each run will last 16 to 30 min depending on the participant's response time. The total duration of rehabilitation sessions will be five weeks. Experiments are controlled by a desktop PC.

Only the intervention group will undergo the rehabilitation and control group will not be informed about details of the intervention study procedure.

Speech stimulus process

The TFS speech consists of single syllable recorded in / a / C / a / with various 16 consonant format which included Aja, Aka, Ara,…. and it will be pronounced by a native-speaking man. The analogue signals will be converted to digital a 16-bit at 44.1 KHz sampling frequency. The stimulus synthesis process will be performed in MATLAB software and the software will be provided in C programming language.

The original bandpass will be filtered into 16 bands of equal bandwidth on a log frequency scale spanning 80 to 8020 Hz. Each Bandpass signal will be decomposed to ENV and TFS by Hilbert transform. The ENV component will be discarded and TFS component will be normalized and TFS component in each band summed lastly creating TFS speech.

After rehabilitation, the SNR (50%) using the WIN[AAD35]  test, and a binaural TFS test and SSQ questionnaire will be evaluated again in intervention group. Results will be compared in intervention and control groups before and after the rehabilitation program.

After rehabilitation, the SNR (50%) using the WIN test, and a binaural TFS test and SSQ questionnaire will be evaluated again. Results will be compared in intervention and control groups before and after the rehabilitation program.[AAD36]

***Outcomes***. *SNR (50%):* single syllable words in the presence of noise at different signal-to-noise ratios (0, + 4, + 8, + 12, + 16, + 20, + 24) as binaural in two study groups and compare the SNR (50%). Differences in scores before and after rehabilitation training between the two groups will be compared.

The Words-in-Noise (WIN) materials were developed to evaluate the ability of listeners to understand words in multitalker babble. The WIN involves in which the level of the noise is fixed and five words are presented at seven signal-to-noise ratios from 24 to 0 dB in 4 dB decrements. The 35 words are spoken by a native male speaker. The metric of interest is the signal-to-noise ratio (S/N) at which recognition performance is 50%, which is a value determined with the Spearman Karber equation see ( *Extended data:* S2).

 Binaural TFS test: determines the binaural change of phase difference at different frequencies in intervention and control groups before and after rehabilitation training.

The correlation between the results of speech perception test scores in the presence of noise with the results of binaural TFS test in the two groups after rehabilitation program will be assessed.

*SSQ questionnaire score:* provided to each group before and after rehabilitation training. Scores between intervention and control groups will be compared ( *Extended data:* S3).

Statistical analysis

In descriptive analysis of data, central tendency and dispersion indices (mean, median and standard deviation) will be used. Kolmogorov–Smirnov test will be used to test whether two random samples are drawn from the same normal distribution. Otherwise its nonparametric equivalent will be used. Depending on the circumstances, paired t-test and analysis of covariance will be used to compare pre- and post-rehabilitation program. Other analytical tests will be used as required during the data processing phase. SPSS software (V20.0, IBM Corporation, New York, USA) will be used for statistical data analysis and the significance level for all tests will be 0.05.

Dissemination

The results of our research will be disseminated through presentations at regional and national audiology conferences. The study outcomes will be published through peer-reviewed journals. There is no limit in the publication of the trial results.[AAD37]

Monitoring

Eight independent audiologists expert [AAD38] who are the academic members of rehabilitation schools in Shahid Beheshti University of Medical Sciences (SBMU) and Iran University of Medical Sciences (IUMS) will monitor patient safety and treatment efficacy. They approved the relevance, clarity and simplicity material of the study.

Study status

The enrolment of the patients has been performed and the allocation will be performed in the near future. The study started in November 2019 and will continue until December 2020.

Discussion[AAD39]

Elderly populations are growing rapidly worldwide, and this higher number of older individuals is associated with an increase in prevalence and incidence of age-related disorders. Age-related hearing loss (presbycusis) is one the most common disorders with an increase in age. Speech perception in noisy environments is very serious difficulty with presbycusis, which can impact negatively on the quality of life of individuals. Loss of speech perception in noisy environments with presbycusis is mostly caused by damage of processing of TFS information. In this study, we will attempt to prove by special rehabilitation training based on TFS damage that age-related hearing loss can be re-established.





[AAD1]effects



[AAD2]Good study! This paper has a potential to add to our current knowledge of presbycusis!



[AAD3]loss of speech perception in noise is not considered as a pathophysiologic reason for presbycusis. It is a functional consequence of presbycusis. Pathophysiology can be due to loss of hair cells etc. Please change accordingly.



[AAD4]was



[AAD5]dB HL



[AAD6]dB HL



[AAD7]nervous



[AAD8]were



[AAD9]change all of these from future tense to past tense. The study has been completed so it can be stated in past tense.



[AAD10]You are missing the results section. Please put your results and findings before the conclusions.



[AAD11]Did you prove it? Did your rehabilitation changed or caused outcomes? Did the participants improve their speech recognition in noise. All of theses are your results and should be expanded and explained.



[AAD12]Restoration? Improvement?



[AAD13]Remove comma



[AAD14]The funding agency



[AAD15]Need to spell out the ENV here as it is the first place it shows up in the article.



[AAD16]Same as above



[AAD17]Tasks of



[AAD18]Not necessary, remove please.



[AAD19]Hypothesized



[AAD20]spelling



[AAD21]verbally



[AAD22]in writing



[AAD23]procedures



[AAD24]were



[AAD25]dB HL, please change in the rest of the manuscript



[AAD26]interaural



[AAD27]spell this out please



[AAD28]was based on



[AAD29]This study has been completed, right? Then you should change the whole article to past tense.



[AAD30]Participants were informed that they can withdraw …



[AAD31]What does this mean? I guess you mean they will be confidentially and anonymously saved.



[AAD32]Spell out this please.



[AAD33]Each? Every?



[AAD34]choose



[AAD35]spell this out please.



[AAD36]this paragraph is repeated above.



[AAD37]Remove. Not necessary



[AAD38]Audiology experts



[AAD39]This section is too short and it needs to be expanded.

Is the study design appropriate for the research question?

Yes

Is the rationale for, and objectives of, the study clearly described?

Yes

Are sufficient details of the methods provided to allow replication by others?

Partly

Are the datasets clearly presented in a useable and accessible format?

Partly

Reviewer Expertise:

Audiology, Auditory Neuroscience and Auditory Electrophysiology

I confirm that I have read this submission and believe that I have an appropriate level of expertise to confirm that it is of an acceptable scientific standard, however I have significant reservations, as outlined above.

# Response

**Published:** 2020-08-09

**Authors:** Parisa Rasouli Fard

**Affiliations:** Department of Audiology, School of Rehabilitation Sciences, Iran University of Medical Sciences. Tehran, Iran, Iran

Dear Dr. Danesh

 Thank you very much for your positive response to our paper. We have now carefully evaluated your comments and revised the manuscript. We hope the new version of the manuscript will be acceptable.

 In the new version of the study protocol we changed most of your very important comments as you recommended.

 We started to write the protocol before to enroll the participants but it is ongoing study now. We changed several sentences from future to past or present.

 The results of our study protocol are not completed yet but primary results indicated speech in noise was improved significantly after rehabilitation training program.

 In the new version the discussion section was expanded and became more adequate.  

 Best regards

# Peer review: Reviewer response for version 1

**DOI:** 10.5256/f1000research.25755.r67180

**Published:** 2020-07-21

**Authors:** Suna Tokgoz‐Yilmaz

**Affiliations:** 1 Diagnosis‐Rehabilitation Center of Hearing, Balance and Speech‐Language Disorders, Ankara University School of Medicine, Ankara, Turkey

The study is excellent, but How to do TFS rehabilitation training for 5 weeks is not detailed. 

- In the study status there was "The enrollment of the patients has been performed and the allocation will be performed in the near future. The study started in November 2019 and will continue until December 2020." But looking at the dates, the work should be finished, but in many sentences, the future tense is used.
- In the inclusion criteria there was "In a preliminary interview, speech perception difficulty will be evaluated with a question if they had difficulty in understanding speech in noise." ​​​For this criteria please look at this article "The role of the medial olivocochlear system in the complaints of understanding speech in noisy environments by individuals with normal hearing" ^1^.
- The discussion and results sections are insufficient.

Is the study design appropriate for the research question?

Yes

Is the rationale for, and objectives of, the study clearly described?

Yes

Are sufficient details of the methods provided to allow replication by others?

Partly

Are the datasets clearly presented in a useable and accessible format?

Partly

Reviewer Expertise:

Audiology

I confirm that I have read this submission and believe that I have an appropriate level of expertise to confirm that it is of an acceptable scientific standard, however I have significant reservations, as outlined above.

## References

[1] : The role of the medial olivocochlear system in the complaints of understanding speech in noisy environments by individuals with normal hearing. *Auris Nasus Larynx*.2013;40(6) : 10.1016/j.anl.2013.04.003 521-4 10.1016/j.anl.2013.04.003 23694738

# Response

**Published:** 2020-07-30

**Authors:** Parisa Rasouli Fard

**Affiliations:** Department of Audiology, School of Rehabilitation Sciences, Iran University of Medical Sciences. Tehran, Iran, Iran

Dear Dr. Tokgoz-Yilmaz,

 Thank you very much for your positive response to our paper. We have now carefully evaluated your comments and revised the manuscript. Below we have specified in detail our responses. We hope you will find the new version of the manuscript acceptable for publication,

**The study is excellent, but How to do TFS rehabilitation training for 5 weeks is not detailed.**

***Respons**: The TFS rehabilitation trainings is performed in our clinic. Rehabilitation sessions are three times a week and 30 minutes each session. However later a considerable number of participants need more than 30 minutes in each session. The total duration of rehabilitation sessions will be five weeks.*

**In the study status there was "The enrollment of the patients has been performed and the allocation will be performed in the near future. The study started in November 2019 and will continue until December 2020." But looking at the dates, the work should be finished, but in many sentences, the future tense is used.**

***Respons:** Thank you for very important comment. We started to write the protocol before to enroll the participants   but it is ongoing study now. We changed several sentences from future to past or present.*

**In the inclusion criteria there was "In a preliminary interview, speech perception difficulty will be evaluated with a question if they had difficulty in understanding speech in noise." ​​​For this criteria please look at this article "The role of the medial olivocochlear system in the complaints of understanding speech in noisy environments by individuals with normal hearing.**

***Respons:** It is very interesting article. It is defined very excellent how   participants complaint of speech understanding in noise by asking seven questions. The population in this study had normal hearing but our subjects had mild to moderate hearing loss due to presbycusis.  However we will use the questionnaire about difficulty to speech understanding in noise in this study in  our population too. We mentioned the study by Tokgoz-Yilmaz et al as a reference.*

**The discussion and results sections are insufficient.**

***Response: **This is study protocol and results of our study are not completed yet.  Primary results are shown rehabilitation training associated with significantly better test results of SNR (50%) by WIN test, binaural TFS test and SSQ questionnaire. The completed results will be published.*

 As reviewer demanded we changed the discussion part to become more sufficient. 

# Peer review: Reviewer response for version 1

**DOI:** 10.5256/f1000research.25755.r64606

**Published:** 2020-06-23

**Authors:** Mojtaba Tavakoli

**Affiliations:** 1 Department of Audiology, School of Rehabilitation, Ahvaz Jundishapur University of Medical Sciences, Ahvaz, Iran

Thanks for the valuable article posted.

 The selection of cases is well done and the research path is well described. Given the problems of people with hearing loss with more falls, please explain if the results are positive, is it possible to do this protocol in more hearing loss or not?

 In the section on formal validity and reliability, please indicate the number obtained from the information collected from eight academic members of the university.

 In the future perspective of this protocol, if the results are positive, it is appropriate to have auditory rehabilitation in the control group. Please explain why the degree of education must be at least a diploma?

Is the study design appropriate for the research question?

Yes

Is the rationale for, and objectives of, the study clearly described?

Yes

Are sufficient details of the methods provided to allow replication by others?

Yes

Are the datasets clearly presented in a useable and accessible format?

Yes

Reviewer Expertise:

Hearing aid

I confirm that I have read this submission and believe that I have an appropriate level of expertise to confirm that it is of an acceptable scientific standard.

# Response

**Published:** 2020-06-25

**Authors:** Parisa Rasouli Fard

**Affiliations:** Department of Audiology, School of Rehabilitation Sciences, Iran University of Medical Sciences. Tehran, Iran, Iran

Dear Reviewer,

 Thank you very much for very important comments and suggestions.  

 In our study the primary results is very positive and participants in intervention group have significantly better speech perception in noisy environments. We will perform rehabilitation training program in subjects with other causes of hearing loss like noise related hearing loss (NRHI).

 The Waltz & Bausell method is used to examine validity of the test. The stimulus was sent to eight independent audiologists expert. They assessed relevance, clarity and simplicity of the test by Likert scale from one (non-relevant, non-simple and non-clarity)   to four scale (complete-relevant, complete-simple and complete-clarity) in each item.The analyze of their evaluation showed content validity index (CVI) 87% validity for the test.

 In this study to increase homogeneity of our study population we decided to eliminate education as nuisance variable (unwanted variable) (1). All participants had to have at least high school diploma which it is more applicable to evaluate the difficulty of test by participants. In future to increase the power of the study and higher external validity the study population with education degree less than high school diploma will be included.

 It is very interesting suggestion, we will perform the test auditory rehabilitation in the control group.  

 1.      Fregni F et al, Critical Thinking in Clinical Research, Oxford University Press, 2018.
